# Comparative Characterization of CpCDPK1 and CpCDPK9, Two Potential Drug Targets against Cryptosporidiosis

**DOI:** 10.3390/microorganisms10020333

**Published:** 2022-02-01

**Authors:** Jiayuan Su, Yiting Shen, Na Li, Yu Li, Ziding Zhang, Lihua Xiao, Yaqiong Guo, Yaoyu Feng

**Affiliations:** 1 State Key Laboratory of Bioreactor Engineering, School of Resource and Environmental Engineering, East China University of Science and Technology, Shanghai 200237, China; sasuke963@sina.com (J.S.); a1272197823@163.com (Y.S.); 2 Center for Emerging and Zoonotic Diseases, College of Veterinary Medicine, South China Agricultural University, Guangzhou 510642, China; nli@scau.edu.cn (N.L.); lxiao@scau.edu.cn (L.X.); 3 Guangdong Laboratory for Lingnan Modern Agriculture, Guangzhou 510642, China; 4 State Key Laboratory of Agrobiotechnology, College of Biological Sciences, China Agricultural University, Beijing 100193, China; yu_li_protein@cau.edu.cn (Y.L.); zidingzhang@cau.edu.cn (Z.Z.)

**Keywords:** *Cryptosporidium parvum*, calcium-dependent protein kinase, protein expression, invasion, inhibitor

## Abstract

As the invasion, egress, and growth of *Cryptosporidium* spp. are regulated by the calcium ion, calcium-dependent protein kinases (CDPKs) are considered potential drug targets against these pathogens. In this study, we expressed CpCDPK1 of *Cryptosporidium parvum* encoded by the cgd3_920 gene and CpCDPK9 encoded by the the cgd7_1260 gene in *Escherichia coli*, and we conducted some comparative studies with quantitative PCR, immunofluorescence staining, and in vitro neutralization assays. By immunofluorescence microscopy, CpCDPK1 was expressed over the entirety of the sporozoites, while CpCDPK9 was mainly expressed in the apical region. The expression of the cgd3_920 gene was the highest at 12 h of the in vitro culture, whereas the expression of the cgd7_1260 gene peaked between 2 h and 6 h. Polyclonal antibodies against these two CpCDPK proteins had similar neutralization efficiency on *C. parvum* growth, reaching approximately 40%. Of the 50 candidate compounds from the molecular docking of CpCDPK1, 10 had significant in vitro anti-cryptosporidial effects, but only one inhibited enzyme activity. For CpCDPK9, five of the forty-five candidate compounds showed significant in vitro anti-cryptosporidial effects. Results obtained from this study suggest that CpCDPK1 and CpCDPK9 might function differently in *C. parvum* infection.

## 1. Introduction

*Cryptosporidium* spp. are apicomplexan parasites responsible for cryptosporidiosis, an important diarrheal disease in humans [1]. In most immunocompetent individuals, the diarrhea lasts for 1–2 weeks [2]. However, cryptosporidiosis can lead to prolonged, life-threatening diarrhea in immunocompromised persons, such as AIDS patients. Among the over 40 established *Cryptosporidium* species, *C. parvum* and *C. hominis* are the dominant *Cryptosporidium* species responsible for human cryptosporidiosis [3]. As *C. parvum* can also infect farm animals, it is a zoonotic pathogen.

There are few effective drugs against cryptosporidiosis. The only one approved by the U.S. Food and Drug Administration is nitazoxanide. Nitazoxanide, however, is not effective in treating cryptosporidiosis in immunocompromised patients or malnourished children, probably because it cannot block the infection [4]. Thus, the development of anti-parasitic drugs is urgently needed for cryptosporidiosis.

Calcium ion flux is a secondary messenger regulating the secretion of apical organelles in apicomplexan parasites [5,6]. Calcium-dependent proteins kinases (CDPKs), as effectors of calcium ion flux, can respond to calcium by means of large-scale intramolecular rearrangement [7]. CDPKs have been identified in plants, ciliates, and apicomplexans, but they are absent in fungi and animals [8]. When the level of calcium increases, the calmodulin-like domain binds calcium, leading to conformational changes in CDPKs that expose the kinase domain from an inactive conformation. 

CDPKs are involved in regulating the lifecycle of parasites [5]. For example, TgCDPK1 of *Toxoplasma gondii* plays a critical role in parasite invasion and egress by controlling the exocytosis of micronemes [9], while TgCDPK3 is involved in the egress of parasites from host cells. CpCDPKs in *C. parvum* are considered to have functions similar to those of their counterparts in other apicomplexans. Moreover, CDPKs are regarded as attractive targets for drug development against apicomplexan parasites, including *Cryptosporidium* spp., due to the absence of homologs in mammals [10]. Among the known CDPKs, CDPK1 is an ideal target for drug design due to its unique amino acid structure [9]. 

The small *Cryptosporidium* genome has an array of genes encoding CDPKs. As most other genes in *Cryptosporidium* spp. are single-copied, the multicopy name of CDPK genes illustrates the importance of CDPKs in the biology of the pathogen. Thus far, several CDPKs of *C. parvum* (CpCDPKs) have been characterized [6,11,12,13]. Among them, CpCDPK1 has been most studied, with several inhibitors of the enzyme being identified [14,15,16]. In this study, we expressed the recombinant proteins of CpCDPK1 encoded by the cgd3_920 gene and of CpCDPK9 encoded by cgd7_1260 gene, and we conducted comparative characterizations of the functions of these two CDPKs. CpCDPK1 is a typical member in the CDPK family, while CpCDPK9 is unique in containing an additional N-terminal EF hand followed by two kinase domains and four C-terminal EF hands. Through immunofluorescence microscopy, quantitative analysis of gene expression, and in vitro neutralization, we examined the potential roles of the two CDPK proteins in host-cell invasion and parasite growth. We further evaluated the anti-*Cryptosporidium* activity of 50 candidate compounds from a molecular docking of CpCDPK1 and their effects on the enzyme activities.

## 2. Materials and Methods 

### 2.1. Cryptosporidium parvum Isolate and Invasion Assay

Oocysts of the *C. parvum* IOWA isolate used in the study were purchased from Waterborne, Inc. (New Orleans, LA, USA) and stored in antibiotics (200 U/mL penicillin, 200 µg/mL streptomycin, and 0.5 µg/mL amphotericin B) at 4 °C. Oocysts were used within 3 months of purchase. They were treated on ice for 10 min with 0.5% sodium hypochlorite prior to the infection of cells. When necessary, the oocysts were further treated with 0.75% sodium taurocholate and 0.25% trypsin at 37 °C for 1 h to obtain free sporozoites. Human ileocecal adenocarcinoma HCT-8 cells were purchased from the Shanghai Branch of the Chinese Academy of Sciences (Shanghai, China) and used in the *Cryptosporidium* invasion assays. In this study, 96-well and 12-well culture plates were used. Cells were seeded into plates and cultured in an Gibco^®^ RPMI 1640 medium (Thermo Fisher Scientific Inc, Waltham, MA USA) containing 10% Gibco^®^ fetal bovine serum (Thermo Fisher Scientific Inc, Waltham, MA USA) at 37 °C in a humidified incubator containing 5% CO_2_ until ~80% confluence. Sodium hypochlorite-treated oocysts in an RPMI 1640 containing 2% fetal bovine serum (Thermo Fisher Scientific Inc, Waltham, MA USA) were inoculated into the culture. After incubation at 37 °C for 2 h, unexcysted oocysts and free sporozoites were washed off with sterile PBS. Then, cells were incubated with a fresh medium containing 2% fetal bovine serum for specified durations.

### 2.2. Construction of Recombinant Plasmid

The nucleotide sequences of the cgd3_920 (XM_001388059.1) and cgd7_1260 (XM_628309.1) genes were retrieved from CryptoDB (http://cryptodb.org; accessed on 12 December 2019). As CpCDPK genes contain no introns, genomic DNA was used directly as a template to amplify the target genes. The template DNA was extracted from the *C. parvum* oocysts using the Qiagen DNeasy Blood & Tissue Kit (Qiagen, Germantown, MD, USA). The coding region was amplified using PCR with the following primers incorporated with restriction sites: cgd3_920-F (5′-CCGGAATTCATGGGAAATACTGCAGTAGGG-3′, including an underlined *EcoR*I restriction site) and cgd3_920-R (5′- CCGCTCGAGCCTGACAAAATTCTGAAGCA-3′, including an underlined *Xho*I restriction site) for the cgd3_920 gene, and cgd7_1270-F (5’-CGAGCTCAAAATGACAGTAGCTACC-3’, including an underlined *Sac*I restriction site) and cgd7_1270-R (5’-CCGCTCGAGATCTAGTTGGCTTG-3’, including an underlined *Xho*I restriction site) for the cgd7_1270 gene. The PCR amplification was conducted with an initial denaturation at 95 °C for 5 min; 35 cycles of amplification at 95 °C for 45 s, 55 °C for 45 s, and 72 °C for 2 min; and a final extension at 72 °C for 7 min. The PCR products were examined using agarose electrophoresis and purified using the Column PCR Product Purification Kit (Qiagen, Germantown, MD, USA). The purified products were digested and ligated into double-digested vector pET28a (Novagen, Darmstadt, Germany). The recombinant plasmids were transformed into *Escherichia coli* DH5α (Tiangen, Beijing, China). Positive colonies were selected from cultures on solid Luria–Bertani (LB) agar containing 50 μg/mL kanamycin and identified by PCR using the T7/T7t universal primers. DNA sequence analysis was used to verify the identity of each clone.

### 2.3. Expression of Recombinant CpCDPKs in E. coli 

Recombinant plasmids containing cgd3_920 and cgd7_1260 sequences were extracted from *E. coli* DH5α using the Plasmid Mini Kit (Qiagen, Germantown, MD, USA) and transformed into *E. coli* BL21 (DE3) (Tiangen, Beijing, China) and *E. coli* Rosetta (Tiangen, Beijing, China), respectively. *E. coli* BL21 cells were incubated at 37 °C in a liquid LB medium, supplemented with 50 μg/mL kanamycin, while *E. coli* Rosetta cells were cultured with an additional 34 μg/mL chloramphenicol. When the OD_600_ reached 0.6, 0.1 mM isopropyl b-D-1-thiogalactopyranoside (IPTG) (Merck, Darmstadt, Germany) was added to induce protein expression at 20 °C overnight. The protein expression was assessed using sodium dodecyl sulfate polyacrylamide gel electrophoresis (SDS–PAGE) and Western blot analyses.

### 2.4. Purification of CDPKs

The recombinant CpCDPK1 expressed in the *E. coli* was purified using Ni-NTA Beads (Qiagen, Germantown, MD, USA) and manufacturer-recommended procedures. For the purification of the recombinant CpCDPK9 expressed in inclusion bodies in *E. coli*, an additional urea treatment was added to dissolve the pellet prior to the purification with the Ni-NTA beads. The purity of proteins was evaluated by SDS–PAGE, while the identity of proteins was assessed using Matrix-Assisted Laser Desorption/Ionization Time of Flight Mass Spectrometry on AB SCIEX TripleTOF 5600 (AB Sciex, MA, USA).

### 2.5. Assessment of Enzymatic Activity of Recombinant CDPKs

The enzymatic activity of the recombinant CpCDPKs was measured using a coupled enzyme ATPase assay as described previously [17]. This assay system contained 150 μM NADH, 300 μM phosphoenolpyruvic acid, and a 3 U/mL mixture of pyruvate kinase and lactate dehydrogenase. Different concentrations (25 μM to 200 μM) of Syntide-2 peptide were used to determine the K_m_ value of recombinant CpCDPKs. The buffer used contained 20 mM HEPES (pH 7.5), 30 mM NaCl, 10 mM MgCl_2_, 1 mM CaCl_2_, 2 μg/mL BSA (Sigma-Aldrich, St. Louis, MO, USA), 10 mM DTT, and 0.01% Tween 20, and the incubation with 20 nM recombinant proteins was performed at 30 °C for 10 min. The enzymatic reaction was initiated by adding 100 μM ATP, with the absorbance being measured at 340 nm on a microplate reader after 40 min. The IC_50_ was calculated using 50 μM Syntide-2 peptide. The mean values from two independent experiments, each performed with two replicates, were used to calculate the K_m_ and IC_50_ values using Prism GraphPad (Version 7.0). 

### 2.6. Preparation of Anti-CDPK Antibodies and Assessment of Their Reactivity to Native Proteins

Polyclonal antibodies against purified recombinant proteins were generated through the immunization of rabbits by GL Biochem Ltd. (Shanghai, China). Post-immune sera were harvested from the immunized animals after the last immunization. Pre-immune sera were collected and used as negative controls. IgG antibodies were purified from the immune sera using beads conjugated with recombinant proteins. The quantity of polyclonal antibodies was assessed by enzyme-linked immunosorbent assay (ELISA) and Western blot. The reactivity of anti-CDPK antibodies to native proteins in *C. parvum* sporozoites was assessed by Western blot as described [18], with polyclonal antibodies being used as primary antibodies and horseradish peroxidase (HRP)-conjugated goat anti-rabbit IgG (Yeasen, Shanghai, China) as secondary antibodies. 

### 2.7. Quantitation of CDPK Gene Expression

The expression of the CDPK genes was evaluated using qPCR analysis of RNA from in vitro cultures of *C. parvum*. Total RNA was isolated from *C. parvum*-infected HCT-8 cells at 0, 2, 6, 12, 24, 36, 48, and 72 h, and used in reverse transcription (RT)-qPCR analysis of the cgd3_920 and cgd7_1260 genes on a LightCycler 480 (Roche, Basel, Switzerland) as described previously [19]. The expression of individual CDPK genes was normalized with data from the *C. parvum* 18S rRNA (Cp18S rRNA) expression [20]. Primers Cp18S-qF (5′- CTAGAGATTGGAGGTTGTTCC -3′) and Cp18S-qR (5′- CTCCACCAACTAAGAACGGC -3′) were used for the Cp18S rRNA gene; the amplicon size was 256 bp [21]. Primers cgd3_920-qF (5′- GAGCACCAGTAGATGCCGTA -3′) and cgd3_920-qR (5′- AAAGGTCCCGCTACCACTCT -3′) were used for qPCR analysis of the cgd3_920 gene, with an amplicon size of 176 bp. Similarly, primers cgd7_1260-qF (5′- CCGTTGTTTGGGAAAAAGTC -3′) and cgd7_1260-qR (5′- TAATTCGGAAATGGGCTACG -3′) were used for the analysis of the cgd7_1260 gene, with an amplicon size of 205 bp. The relative levels of gene expression were calculated using threshold cycle (C_T_) values from the qPCR and the approach [22]. Three biological replicates and two technical replicates were used in each experiment. The mean values in this report were obtained from three independent experiments.

### 2.8. Localization of CDPK Protein Expression in Life Cycle Stages

Oocysts and free sporozoites were fixed on slides with 4% paraformaldehyde in PBS for 30 min to examine CpCDPK expression in these stages. For other developmental stages, intracellular parasites in HCT-8 cell cultures harvested 24 and 48 h after infection were fixed with 4% paraformaldehyde and permeabilized with 0.5% Triton X-100. The polyclonal antibodies against CpCDPKs (5.0 μg/mL, diluted in PBS) were used as the primary antibodies, while Alexa Fluor 594-conjugated Goat Anti-rabbit IgG (Cell Signaling Technology, Danvers, MA, USA) was used as the secondary antibody in an immunofluorescence assay. 4’,6-diamidino-2-phenylindole (DAPI, Roche, Basel, Switzerland) was used to counterstain the nuclei of the organisms [23]. After being mounted with the No-Fade Mounting Medium (Boster, Wuhan, China), the slides were covered with coverslips, sealed with nail polish, and examined using the differential interference contrast (DIC) and immunofluorescence microscopy under a BX53 microscope (Olympus, Tokyo, Japan).

### 2.9. In Vitro Neutralization of Sporozoite Invasion

A neutralization assay was used to evaluate the potential role of CDPK proteins during the invasion of *C. parvum* [17]. Oocysts of *C. parvum* IOWA isolate were treated with sodium hypochlorite as described above, and incubated with immune serum or pre-immune serum at 1:100, 1:200, 1:500, and 1:1000 dilutions before infection [19]. Cy3-labeled Sporo-Glo antibodies against *C. parvum* (Waterborne, Inc., New Orleans, LA, USA) were used to monitor the developmental stages as described [24]. The stained culture was examined under a BX53 immunofluorescence microscope. For each coverslip, 50 images were captured randomly under 200 × microscopy. All the images were analyzed using the ImageJ software (https://imagej.nih.gov/ij/; accessed on 21 June 2019). Experiments were performed in triplicate, and data from three independent experiments were compared among treatment groups using the Student’s *t*-test. The neutralization effects of immune sera were normalized using data from cultures treated with pre-immune sera.

#### In Vitro Inhibition of Invasion and Development using Candidate CpCDPK Inhibitors

The inhibition capability of candidate CpCDPK inhibitors in blocking the *C. parvum* in vitro invasion and development was assessed using the in vitro invasion and development assay described above. These small molecules were selected based on the factors of molecular weight, ligand efficiency, coulomb energy, and H-bond energy in molecular docking of CpCDPK1 and CpCDPK9 in the ChemDiv database. They were inoculated into HCT-8 cell cultures together with hypochlorite-treated oocysts. After 2 h of incubation, free sporozoites were washed off, and the culture medium was replaced with RPMI 1640 supplemented with diluted compounds and allowed to incubate for an additional 22 h. Total RNA was isolated from *C. parvum*-infected HCT-8 cells and used as a template in RT-qPCR analysis on a LightCycler 480. The primers used included Cp18S-F (5′- TTGTTCCTTACTCCTTCAGCAC -3′) and Cp18S-R (5′- TCCTTCCTATGTCTGGACCTG -3′) for the 18S rRNA gene of *C. parvum*, and Hs18S-F (5′- GGCGCCCCCTCGATGCTCTTA -3′) and Hs18S-R (5′- CCCCCGGCCGTCCCTCTTA -3′) for the 18S rRNA gene of the host cells [25]. At least two technical replicates were included in each RT-qPCR analysis of the samples. Data from the 18S rRNA gene of the host cells were used in data normalization. The relative parasite loads were calculated using the ΔΔC_T_ approach [25]. 

## 3. Results 

### 3.1. Production of Recombinant CpCDPKs in E. coli

**Figure 1:**
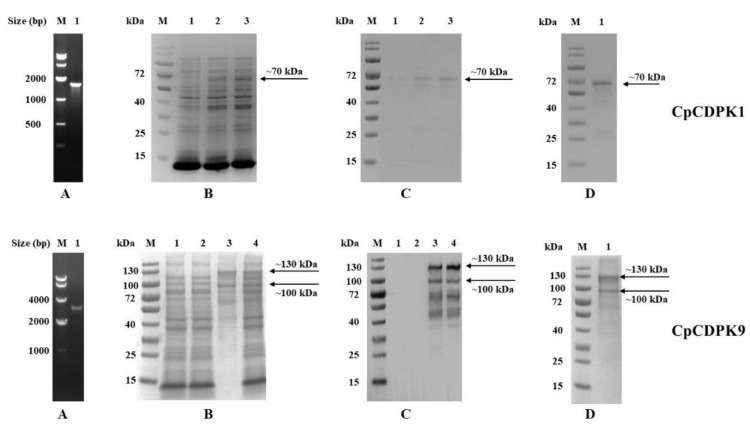
Production and purification of recombinant CDPKs of *Cryptosporidium parvum*. (**A**) PCR amplification of the cgd3_920 (upper panel) and cgd7_1260 (lower panel) genes in genomic DNA. Lane M: molecular markers; lane 1: PCR product. (**B**) Expression of recombinant proteins in *Escherichia coli* BL21 (DE3) as indicated by SDS–PAGE analysis. Upper panel for CpCDPK1: lane M, molecular weight markers; lane 1, lysate from bacterial culture transformed with the recombinant plasmid with no IPTG induction; lane 2, lysate from similar bacteria culture induced with IPTG for 2 h; lane 3, lysate from bacteria culture induced with IPTG for 8 h, with the expected product being indicated by an arrow. Lower panel for CpCDPK9: lane M, molecular weight markers; lane 1, lysate from bacterial culture transformed with the recombinant plasmid with no IPTG induction; lane 2, supernatant of lysate from transformed bacterial culture with IPTG induction; lane 3, pellet of lysate from transformed bacterial culture with IPTG induction; 4, lysate from transformed bacterial culture with IPTG induction, with the expected product being indicated by an arrow. (**C**) Western blot analysis of the recombinant CpCDPK1 (upper panel) and CpCDPK9 (lower panel). Lane labels are the same as in b. (**D**) Purification of recombinant CpCDPK1 (upper panel) and recombinant CpCDPK9 (lower panel). Lane M, molecular weight markers; lane 1, purified recombinant protein from Ni-NTA affinity chromatography.

We successfully cloned the cgd3_920 and cgd7_1260 genes into the pET28a vector (Figure 1A) and verified the identity and accuracy of DNA sequences by DNA sequence analysis. The recombinant CpCDPK1 was expressed successfully at the predicted size of ~70 kDa in *E. coli* BL21 (Figure 1B,C, top panel), while CpCDPK9 was expressed in *E. coli* Rosetta due to the presence of rare codons (Figure 1B,C, bottom panel). Although the expression of CpCDPK9 produced more bands (130 kDa, 100 kDa, 70 kDa, and 40 kDa) than expected, the results of the MALDI–TOF–MS analysis confirmed the CpCDPK9 identity of the extra fragments (data not shown). Pure recombinant CpCDPK1 protein was obtained using Ni-NTA purification through the His-tag incorporated (Figure 1D, top panel), while pure CpCDPK9 protein was obtained by gel extraction (Figure 1D, bottom panel).

### 3.2. Expression of Native CDPKs in Sporozoites

**Figure 2:**
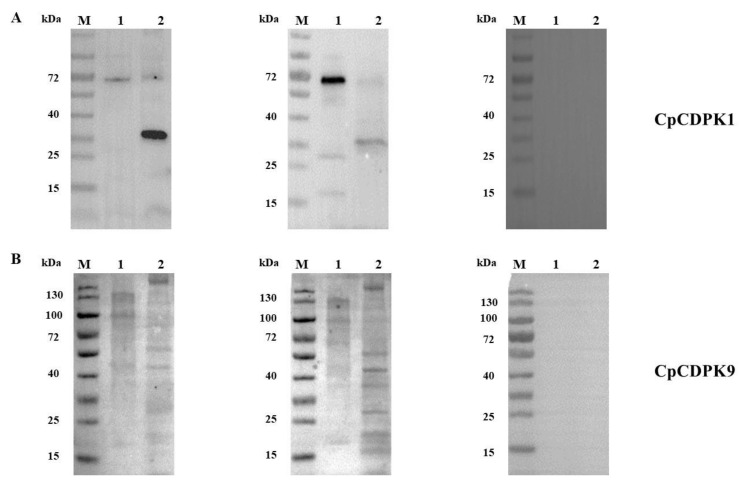
Expression of native CpCDPKs in crude extract of sporozoites of *Cryptosporidium parvum*. (**A**) Western blot analysis of native CpCDPK1 using polyclonal antibodies (left panel), post-immune sera (middle panel), and pre-immune sera (right panel). Lane M, molecular weight markers; lane 1, purified recombinant CpCDPK1; lane 2, crude proteins extracted from sporozoites. (**B**) Western blots analysis of native CpCDPK9 using polyclonal antibodies (left panel), post-immune sera (middle panel), and pre-immune sera (right panel). Lane M, molecular weight markers; lane 1, purified recombinant CpCDPK9; lane 2, crude proteins extracted from sporozoites.

In the analysis of native CDPK proteins in crude extracts of sporozoites, the native CDPK1 was recognized by the anti-CpCDPK1 antibodies, generating two bands of ~30 kDa and ~70 kDa (Figure 2A). In contrast, multiple bands of 130 kDa, 100 kDa, 50 kDa, and 30 kDa in sporozoite extracts were recognized by the anti-CpCDPK9 antibodies (Figure 2B). As expected, these antibodies also recognized their corresponding recombinant proteins, while pre-immune sera had no reaction with either native proteins or recombinant proteins.

### 3.3. CDPK expression in Developmental Stages

**Figure 3:**
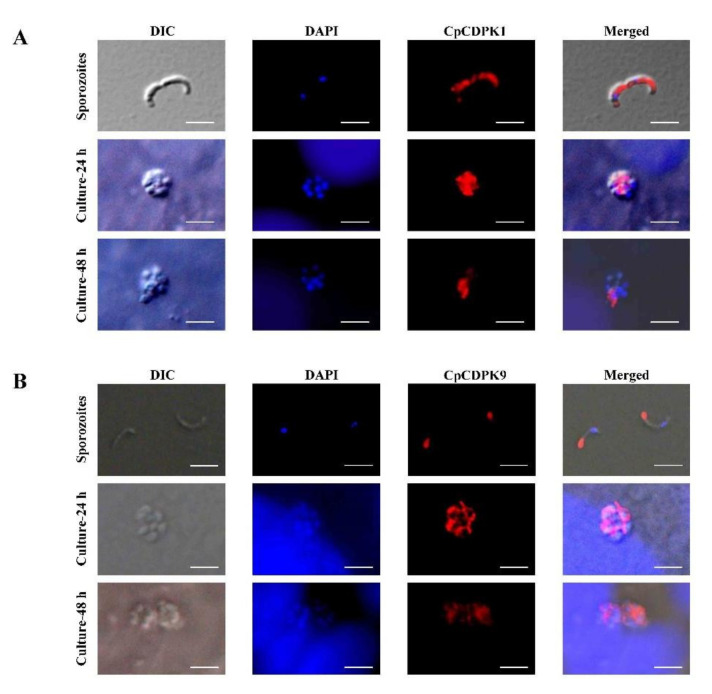
Localization of the expression of CpCDPKs in life cycle stages of *Cryptosporidium parvum*. (**A**) Expression of CpCDPK1 in sporozoites (top panel) and developmental stages in HCT-8 cell cultures at 24 h (middle panel) and at 48 h (bottom panel). The images were taken under differential interference contrast (DIC), with the nuclei being counter-stained with 4ʹ,6-diamidino-2-phenylindole (DAPI), and parasites stained with Alexa 594-labeled CpCDPK1 (CpCDPK1). The merged image is a superimposition of the three images (Merged). Bars = 5 μm. (**B**) Expression of CpCDPK9 in sporozoites (top panel) and intracellular developmental stages of *C. parvum* in HCT-8 cell cultures at 24 h (middle panel) and 48 h (bottom panel). The images were taken under differential interference contrast (DIC), with the nuclei being counter-stained with 4ʹ,6-diamidino-2-phenylindole (DAPI), and parasites stained with Alexa 594-labeled CpCDPK9 (CpCDPK9). The merged image is a superimposition of the three images (Merged). Bars = 5 μm.

In immunofluorescence microscopy, CpCDPK1 antibodies reacted with the entirety of the sporozoites of *C. parvum* (Figure 3A), while no reactivity to *C. parvum* sporozoites was detected using pre-immune sera. In assessment of CpCDPK1 expression in intracellular stages, CpCDPK1 antibodies had diffused reactivities with merozoites within meronts at 24 h of culture. The reactivity was restricted to the part of merozoites opposite to the nuclei at 48 h (Figure 3A).

CpCDPK9 antibodies reacted mostly with the anterior regions of *C. parvum* sporozoites (Figure 3B). At 24 h of the *C. parvum* culture, anti-CpCDPK9 antibodies had high reactivity to the entirety of the merozoites within meronts. At 48 h, the reactivity of the antibodies to the merozoites appeared similar at reduced intensity. 

### 3.4. Expression of CDPK Genes in C. parvum Culture

**Figure 4:**
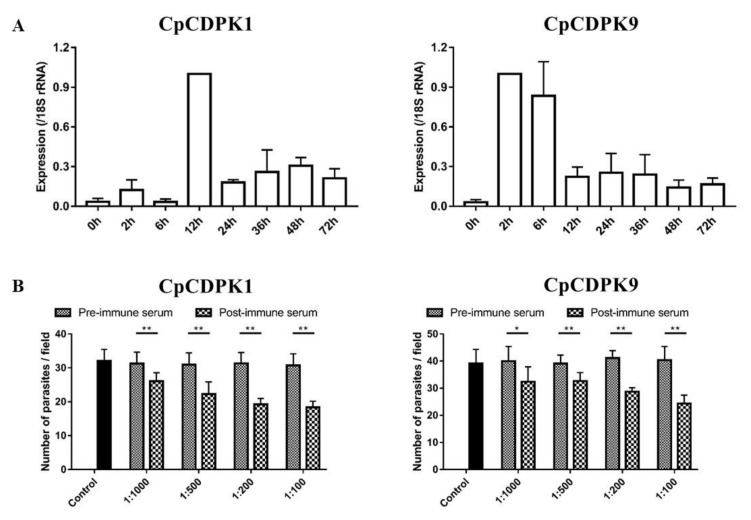
Function studies of CpCDPK1 (left panel) and CpCDPK9 (right panel). (**A**) Expression levels of the cgd3_920 (left panel) and cgd7_1260 (right panel) genes in developmental stages of *C. parvum*. (**B**) Neutralization efficiency of immune sera against CpCDPK1 (left panel) and CpCDPK9 (right panel) on *C. parvum* invasion. Cultures were treated with immune sera or pre-immune sera. Cultures with no addition of rabbit sera served as control. Data presented are mean ± SD from three independent experiments. The significance of differences between treatment groups in neutralization studies of proteins is indicated above the bars (*, *p* < 0.05; **, *p* < 0.01).

The relative expression levels of the CDPK genes during the intracellular development of *C. parvum* in cell culture were evaluated by qPCR. The expression of the cgd3_920 gene appeared low during the first 6 h of culture, reached peak at 12 h, and maintained at low levels thereafter (Figure 4A, left panel). In contrast, the expression of the cgd7_1260 gene was highest at 2 h and 6 h of culture, and maintained at low levels from 12 h to 72 h of culture (Figure 4A, right panel). 

### 3.5. Neutralization of Sporozoite Invasion by CDPK Antibodies

Compared with cultures treated with pre-immune sera, a modest but significant reduction in parasite load in *C. parvum* cultures was seen when sporozoites were pre-incubated with post-immune sera from CpCDPK1-immunized rabbits (Figure 4B, left panel). The inhibition rate was 16.6% (31.3 ± 3.3 and 26.1 ± 2.5 per 200 × field for pre- and post-immune sera, respectively; *t*_(2)_ = 11.365, *p* = 0.008) at 1:1,000 dilution, 28.4% (31.0 ± 3.5 and 22.2 ± 3.6 per 200 × field for pre- and post-immune sera, respectively; *t*_(2)_ = 76.676, *p* = 0.000) at 1:500 dilution, and 38.7% (31.3 ± 3.2 and 19.2 ± 1.8 per 200 × field for pre- and post-immune sera, respectively; *t*_(2)_ = 11.604, *p* = 0.007) at 1:200 dilution, and 40.4% (30.7 ± 3.4 and 18.3 ± 1.7 per 200 × field for pre- and post-immune sera, respectively; *t*_(2)_ = 11.172, *p* = 0.008) at 1:100 dilution. For control cultures with no addition of rabbit sera, the parasite load was 32.0 ± 3.5 per 200 × field. 

A similar inhibition of sporozoite invasion was achieved with anti-CpCDPK9 antibodies. The inhibition rate was 19.8% (40.0 ± 3.12 and 32.1 ± 3.2 per 200 × field for pre- and post-immune sera, respectively; *t*_(2)_ = 18.400, *p* = 0.003) at 1:1,000 dilution, 16.6% (39.1 ± 1.8 and 32.6 ± 1.8 per 200 × field for pre- and post-immune sera, respectively; *t*_(2)_ = 35.874, *p* = 0.001) at 1:500 dilution, and 30.3% (41.2 ± 1.5 and 28.7 ± 0.8 per 200 × field for pre- and post-immune sera, respectively; *t*_(2)_ = 17.003, *p* = 0.003) at 1:200 dilution, and 39.5% (40.3 ± 2.9 and 24.4 ± 1.8 per 200 × field for pre- and post-immune sera, respectively; *t*_(2)_ = 13.932, *p* = 0.005) at 1:100 dilution (Figure 4B, right panel). 

### 3.6. In Vitro Anti-Cryptosporidial Activities of Small Molecule Compounds

**Figure 5:**
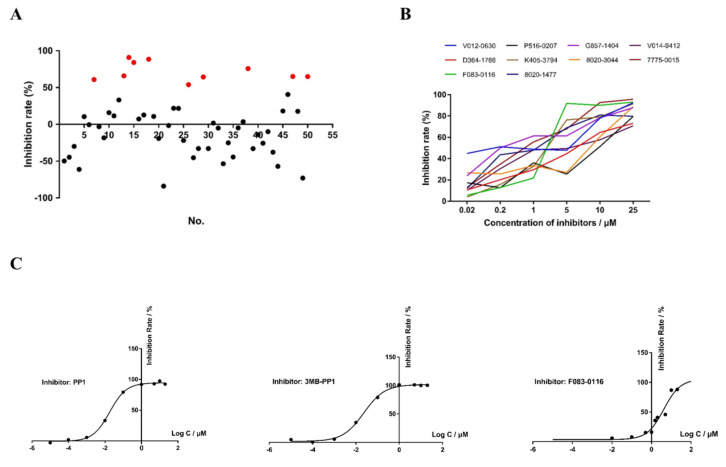
Effects of small-molecule compounds from molecular docking of CpCDPK1 on *Cryptosporidium parvum* in vitro invasion and development and enzyme activity of CpCDPK1. (**A**) Efficacy of all 50 compounds (10 μM each) targeting CpCDPK1 against *C. parvum* invasion based on three independent experiments. Red dots indicate efficacies of over 50%. (**B**) Inhibition efficacy of 10 of the selected compounds in dose-response experiments. (**C**) IC_50_ of candidate CpCDPK1 inhibitor F083-0116 on the enzymatic activity of CpCDPK1, with known inhibitors PP1 and 3MB-PP1 as the positive controls.

In primary evaluations of 50 small-molecule compounds targeting CpCDPK1 (Table 1) by qPCR analysis of parasite loads in a *C. parvum* culture at 10 μM, 10 compounds reduced the parasite load by over 50% (Figure 5A). Some compounds produced high inhibition of *C. parvum* in vitro invasion and development. With the method of the ΔΔCT approach, relative parasite loads were calculated to evaluate the inhibition rate. Meanwhile, we also monitored cell growth by the expression level of the 18S rRNA gene of the host cells. By comparing the difference between the Ct values of the samples with and without the compound, the cell cytotoxicity could be also evaluated. For those candidates that displayed negative inhibition values, they were more likely to have cytotoxicity on the host cells since a decrease in the number of host cells would be observed. For the effective inhibitors mentioned in the report, we did not see any significant difference in host-cell growth after the addition of the compounds. In microscopy observations of the cell culture, we also did not detect any obvious cellular damage. For example, the inhibition rate of compound G857-1404 reached 84.05%. Some candidates displayed negative inhibition values, indicating that those compounds might have cytotoxicity on host cells. In a dose-response experiment at 1 nM to 25 μM concentrations, all 10 compounds had inhibition rates greater than 70% at the concentration of 25 μM (Figure 5B). The EC_50_ values were 3.36 μM, 2.14 μM, 0.10 μM, 1.81 μM, 0.26 μM, 0.24 μM, and 0.66 μM for D364-1766, F083-0116, G857-1404, K405-3794, 8020-1477, V014-9412, and 7775-0015, respectively. Due to the abnormal decay of the inhibitory effects of V012-0630, 8020-3044, and P516-0207, the EC_50_ values of these compounds could not be reliably calculated.

**Figure 6:**
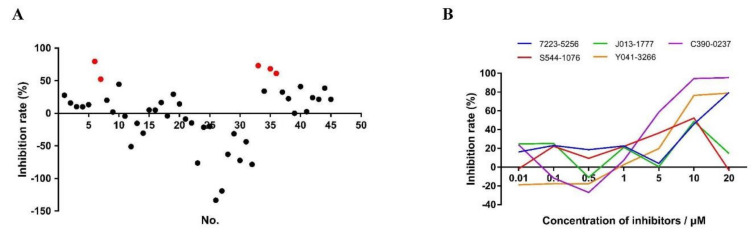
Effects of small-molecule compounds from molecular docking of CpCDPK9 on *Cryptosporidium parvum* invasion. (**A**) Efficacy of all 45 compounds (10 μM each) targeting CpCDPK9 obtained from three independent experiments. Red dots indicate efficacies of over 50%. (**B**) Efficacy of 5 of the selected compounds in dose-response experiments.

We also assessed 45 compounds from molecular docking of CpCDPK9 (Table 2). Five of them, including 7223-5256, J013-1777, C390-0237, S544-1076, and Y041-3266, caused significant reduction (inhibition rate > 50%) against *C. parvum* invasion at the concentration of 10 μM (Figure 6A). The EC_50_ values were 10.04 μM, 4.53 μM, and 5.69 μM for 7223-5256, C390-0237, and Y041-3266, respectively, based on data from the dose-response experiment (Figure 6B). The EC_50_ value for the remaining compounds could not be reliably calculated due to the abnormal decay curve.

### 3.7. Effects of Candidate CDPK Inhibitors on Enzymatic Activities

The Syntide-2-mediated ATPase assay was used to determine the Michaelis constant (K_m_-_Syntide-2_) value of recombinant CpCDPK1. The K_m_ of CpCDPK1 was 283 μM/L using the double-reciprocal method (y = 3787.5 x + 13.372, R^2^ = 0.9994). All the candidate compounds were evaluated in vitro at 10 μM for effects on the enzyme activity of CpCDPK1, with PP1 and 3MB-PP1 as the positive controls. Among them, only F083-0116 (8-chloro-*N*-cyclopentyl-5-oxo-1-thioxo-4,5-dihydro-1*H*-thiazolo[3,4-*a*]quinazoline-3-arboxamide) showed inhibition of greater than 50%. In further dose-response evaluation, the IC50 of PP1, 3MB-PP1, and F083-0116 were 0.018 μM, 0.023 μM, and 4.315 μM, respectively (Figure 5C). As the recombinant CpCDPK9 had no enzymatic activities, the effect of these candidate compounds was not assessed further.

## 4. Discussion

Results of this study suggest that CpCDPK1 and CpCDPK9 might play different roles in the invasion and growth of *C. parvum*. They have different sizes and domain structures, are transcribed at different times during development, and are likely expressed in different organelles of the pathogen. Nevertheless, both CDPKs could be important to the development of *C. parvum*, as additions of antibodies or candidate inhibitors against CpCDPK1 and CpCDPK9 to the in vitro cultures’ reduced parasite load.

The two CpCDPKs examined in the present study differ in predicted functional domains and expression patterns. CDPKs contain EF-hand motifs attached to the C-terminus of a kinase domain [26] and are activated by calcium ions, which is an important signal-transporting messenger [27]. Canonical CDPK proteins, such as the 70-kDa CpCDPK1, have four EF hands at the C-terminus. Therefore, the presence of additional N-terminal EF domains in CpCDPK9, predicted to be 130 kDa, is unusual [28]. The expression of these two CDPKs in native proteins appears to be different. In the present study, CpCDPK1 was shown to exist at a predicted size of ~70 kDa and a truncated form of ~30 kDa in *C. parvum* sporozoite extracts. As CDPK sequences contain proteolytic cleave sites, they are probably proteolytically processed in *C. parvum*. In previous studies, a pro-domain cleave site, with the form of SΦXE/D (in which Φ is hydrophobic and X is any amino acid), has been observed in toxolysin-1 [29,30]. CDPK1 has three such sites, with the sequence SFGE in amino acids 86-89, SAID in amino acids 458-461, and SIIE in amino acids 509-512. Therefore, the truncated form of 30 kDa in sporozoite extracts might be a proteolytic product. Proteolytic processing apparently also occurs in CpCDPK9, as seen in antibody reactivities to both native and recombinant CpCDPK9 in this study. CDPK9 contains more proteolytic cleave sites than CDPK1, which could have contributed to multiple products in the SDS–PAGE analysis of the native and recombinant proteins. Taken together, it is likely that CDPKs are post-translationally processed to exert their biological functions.

Data generated in the study support previous observations on the importance of CpCDPK1 in the growth of *C. parvum*. The CDPKs in *Cryptosporidium* have high phylogenetic homology to their counterparts in *T. gondii* and *P. falciparum* [13]. The similarity of these homologous enzymes indicates that CpCDPKs could play important roles in the invasion and growth of *C. parvum* similar to other apicomplexans [6]. CpCDPK1 is highly homologous to TgCDPK1, which was shown to be essential in host-cell attachment and invasion [31]. Data from the present study suggest that CpCDPK1 is expressed on both sporozoites and merozoites of *C. parvum*. In neutralization experiments, polyclonal antibodies against CpCDPK1 reduced the parasite load by nearly 40%. These results support the recent observation that *Cp*CDPK1 is essential in the development of *C. parvum* [15].

CpCDPK9 also appears to be involved in the development of *C. parvum*. Although belonging to the same family, CpCDPK9 has an expression pattern significantly different from CpCDPK1. Although these two CDPKs are both expressed on sporozoites and merozoites, CpCDPK1 is expressed over the entirety of *C. parvum* sporozoites, while CpCDPK9 is concentrated in the anterior regions of sporozoites. It has been reported that most invasion-associated proteins are located in the apical regions of sporozoites [32]. Therefore, CpCDPK9 is more likely to play a role in the invasion of *C. parvum*, while CpCDPK1 might participate in the growth of the pathogen. In neutralization experiments, anti-CpCDPK9 antibodies reduced the invasion by nearly 40%. The reduction is moderate, which might be due to the multiple strategies used by *Cryptosporidium* to invade host cells [33]. In addition, the peak expression of the gene encoding CDPK9 at 2 h to 6 h of in vitro cultivation of the pathogen supports the role of the protein in invasion. Considering that the calcium flux is the second messenger regulating the exocytosis process, CpCDPK9 expressed in apical region protein could participate in the invasion of host cells and early development of *C. parvum*. In other apicomplexan parasites, different CDPKs have been suggested as playing overlapping and distinct roles in invasion and growth [34].

The specific inhibition of the CDPK activity could be useful in the development of potential treatments against *C. parvum* infection. In the present study, compound F083-0116 inhibited the enzyme activity of recombinant CpCDPK1 and blocked *C. parvum* growth significantly in vitro. Although previous observations suggested there was no apparent correlation between anti-CpCDPK1 activities and *C. parvum* growth-inhibition by candidate CDPK1 inhibitors [17,35], we have provided some evidence suggesting that inhibiting the activity of CpCDPK1 can potentially affect the invasion of *C. parvum*. The compound F083-0116 can also inhibit the activity of CpCDPK3, which is phylogenetically related to CpCDPK1 and has similar domain structures [12]. As *C. parvum* development cannot be inhibited by anti-CpCDPK3 antibodies, the reduction of *C. parvum* growth by compound F083-0116 could be mediated through CpCDPK1 rather than CpCDPK3. This agrees with the data from a recent gene depletion study that CpCDPK1 is essential in the development of *C. parvum* [15].

## 5. Conclusions

We have conducted preliminary characterizations of two CDPKs in *Cryptosporidium* and obtained some evidence indicating that CpCDPK proteins play important roles in *Cryptosporidium* development. However, more biological and immuno-histological studies, such as the use of electron microscopy to examine the subcellular localization and the genetic modifications of the pathogen by CRISPR–Cas9, are needed to further understand the functions of CpCDPKs. We found some compounds can block the *C. parvum* growth significantly in vitro. However, the candidate CpCDPK inhibitors identified need further, thorough evaluations of the effectiveness and action mechanisms. The identification of these inhibitors can improve the understanding of the function of CDPKs in *Cryptosporidium* spp. and promote the development of better therapeutic agents against cryptosporidiosis.

## Figures and Tables

**Table 1:** Candidate Compounds Selected for In Vitro Evaluation of Effects on *Cryptosporidium* Growth Based on Molecular Docking of CpCDPK1.

No.	Compound	No.	Compound	No.	Compound
1	D008-0179	18	8020-3044	35	C499-0205
2	F472-0429	19	8019-3516	36	D730-0071
3	S021-0111	20	G856-6766	37	J106-0712
4	E518-0433	21	F722-0391	38	8020-1477
5	C753-1493	22	8015-7028	39	F470-0515
6	D046-0013	23	J101-0311	40	C118-0100
7	V012-0630	24	T482-1629	41	D126-0439
8	D392-0696	25	S824-0226	42	D006-0238
9	D008-0217	26	P516-0207	43	4514-2069
10	C879-1492	27	5685-0834	44	Y041-7886
11	S021-0065	28	D226-0165	45	3028-4958
12	8012-3948	29	K405-3794	46	7919-0012
13	D364-1766	30	Y020-1235	47	V014-9412
14	F083-0116	31	G419-0776	48	P759-1388
15	G857-1404	32	D271-0476	49	D401-0791
16	E455-0304	33	C197-0058	50	7775-0015
17	D426-1054	34	C066-1821		

**Table 2:** Candidate Compounds Selected for In vitro Evaluation of Effects on *Cryptosporidium* Growth Based on Molecular Docking of CpCDPK9.

No.	Compound	No.	Compound	No.	Compound
1	8012-9491	16	S616-8028	31	Y041-3613
2	P896-0008	17	P896-0153	32	C276-1003
3	D271-0053	18	F401-0004	33	7223-5256
4	P937-2651	19	4428-0282	34	M976-0119
5	S641-4525	20	Y041-6010	35	S544-1076
6	C390-0237	21	F722-0985	36	J013-1777
7	Y041-3206	22	2995-0027	37	4358-6015
8	J106-0113	23	D074-0339	38	D126-0082
9	Y020-6058	24	7655-0020	39	8013-1632
10	G639-3793	25	8018-3260	40	D585-0146
11	G072-0423	26	S021-0079	41	S629-0195
12	D021-0194	27	D389-0696	42	Y020-2366
13	P814-5254	28	D330-0081	43	D361-0101
14	E687-0240	29	D718-0573	44	Y021-0940
15	M510-0341	30	J106-0289	45	C200-7683

## Data Availability

All data that support the findings of this study are presented in this article.
